# Impact of salt concentration and free magnesium on human beta-cardiac myosin reveal important details about the conserved mechanochemical mechanism

**DOI:** 10.1016/j.jbc.2026.113114

**Published:** 2026-05-06

**Authors:** Jinghua Ge, Michael R. Ebert, Skylar M.L. Bodt, Satyabrata Majumder, Wen Ma, Christopher M. Yengo

**Affiliations:** 1 Department of Cell and Biological Systems, Penn State College of Medicine, Hershey, Pennsylvania, USA; 2 Department of Physics, University of Vermont, Burlington, Vermont, USA

**Keywords:** actin, ATPase, cardiac, heart failure, motility, myosin

## Abstract

The myosin ATPase cycle is dramatically accelerated by the presence of actin, while the structural details of actin-activation are still unclear. We found that a dilated cardiomyopathy mutation, E525K, in human beta-cardiac myosin subfragment 1 (S1) enhanced actin-activation of phosphate release and lever arm rotation ∼3-fold. We hypothesized that abolishment of the conserved 484 to 525 salt bridge allows for lysine 525 to more readily interact with actin. Indeed, we found that actin-activated ATPase activity of E525K was quite resistant to changes in salt concentration (20 mM to 100 mM KCl) compared to the WT motor, while *in vitro* actin-gliding velocities were similarly altered. Direct measurements of pyrene actin binding revealed that the E525K mutation accelerates attachment to actin. We found that the actin-activated ATPase activity of E525K was less sensitive to magnesium (Mg) than WT, indicating that the mutation allosterically alters the coordination of Mg in the active site. In addition, the Mg dependence of ADP release was significantly faster in the mutant at low Mg. Molecular dynamics simulations demonstrate that E525K increases the conformational variation of the active site in the ADP-bound state. Our results suggest that the E525K mutation enhances actin affinity by accelerating the transition from weak to strong actin binding, which highlights the importance of the activation loop/relay helix communication pathway during actin-activated Pi-release. In addition, E525K alters Mg coordination in the active site further demonstrating the importance of the 484 to 525 salt bridge in allosterically coupling the active site and actin-binding region.

Cytoskeletal motors are capable of using the energy from ATP hydrolysis to produce force and motion through their interaction with a cytoskeletal track, referred to as the mechanochemical cycle (1). Myosin is a member of the P-loop family of ATPases that have a highly conserved ATP-binding pocket which coordinates magnesium-ATP binding and hydrolysis (2). One important feature of the myosin ATPase cycle is that the presence of actin dramatically stimulates the ATPase activity, presumably by accelerating the product release steps (2, 3). Thus, the mechanical and biochemical cycles are closely coordinated to enhance the efficiency of the reaction and to prevent futile ATPase cycles. A key unanswered question about myosin and other cytoskeletal motors is the structural mechanism of how their ATPase cycles are regulated by binding to their associated track.

In a recent study, we examined a mutation (E525K) in beta-cardiac myosin associated with dilated cardiomyopathy and found that it dramatically accelerated the actin-activated ATPase reaction and achieved maximal ATPase at a much lower actin concentration (4, 5). The E525K mutation is located in the activation loop, which is a loop thought to be crucial for triggering the actin binding–induced activation of the ATPase reaction (Fig. 1) (6). However, the details of the allosteric mechanism are currently unclear. For example, the previous work was not able to determine if the activation loop plays a role in the initial attachment to actin that is known to be electrostatic in nature or if it plays a role in the transition into the strongly bound actomyosin complex. In our previous study, we also found that E525K abolished a salt-bridge with K484 in the highly-conserved relay helix, which is an important helix that allows communication between the actin-binding region, active site, and lever arm (Fig. 1) (4). Molecular-modeling analyses suggested that the lysine at the 525 position could sample conformations compatible with electrostatic contacts to actin (4). Thus, in the current study, we tested the hypothesis that the 525 lysine supports electrostatic interactions with actin, presumably since it cannot form a salt-bridge with K484, providing a structural rationale for the enhanced actin affinity.

**Figure 1:**
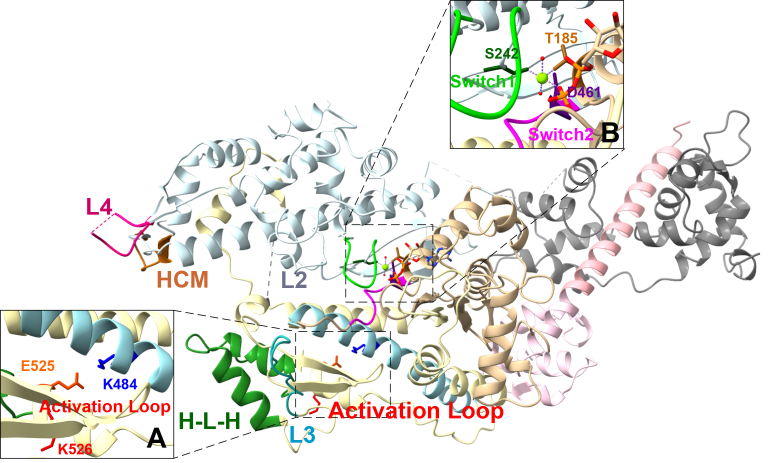
**Mg coordination in the active site and the 484 to 525 salt-bridge.** Close-ups of Mg coordination in the active site and the 484 to 525 salt-bridge and activation loop. The relay helix is displayed in *light blue*. A close up of the active site demonstrates coordination of Mg (residues T185, S242, D461) and the conserved switch I (*green*) and switch 2 (*pink*) regions that coordinate ATP (PDB 5N69).

Another important aspect of P-loop ATPases is their coordination of magnesium (referred to as Mg throughout) in the active site which plays an important role in the mechanochemical cycle (Fig. 1) (7). For example, myosin will still exhibit robust ATPase activity without Mg, but its ATPase cycling becomes uncoupled from force generation (8). However, the structural details of how Mg coordination impacts actin-activated ATPase and the mechanochemical reaction are currently unclear. We found that the E525K mutation alters the free Mg dependence of the ADP release step, which suggests the mutation may alter the allosteric mechanism associated with actin-binding induced changes in the active site that trigger product release (4). Although previous work strongly supports a role for Mg in mediating the ADP release step (9, 10, 11), it is still unclear how Mg can alter other steps in the ATPase cycle including actin binding and phosphate release. In the current study, we examined the impact of free Mg on WT and E525K beta-cardiac myosin to further elucidate how Mg may alter specific steps in the ATPase reaction.

Overall, the current study was designed to probe the structural basis of actin-activation of the myosin ATPase reaction by performing enzymatic, motor, and transient kinetic studies with the WT and E525K beta cardiac myosin motor. Our results provide crucial insight into the role of the activation loop in coordinating actin binding and the ATPase reaction. In addition, our studies reveal new insight into the role of Mg coordination in mediating the myosin ATPase cycle.

## Results

To investigate how electrostatic interactions and divalent cation coordination regulate the enzymatic and mechanical function of human β-cardiac myosin, we tested both WT and a dilated cardiomyopathy-associated mutation, E525K. We hypothesized that E525K alters allosteric communication between the actin-binding interface and the catalytic site, thereby modulating mechanochemical output. To test this, we expressed and purified recombinant beta-cardiac myosin constructs containing either WT or E525K motor domains and performed a series of biochemical and biophysical assays to assess actin-activated steady-state ATPase activity, *in vitro* motility, ADP-release kinetics, and direct measurements of actin binding under controlled salt and free magnesium concentrations. In parallel, we evaluated the structural basis of the impact of E525K on Mg coordination and its potential allosteric effects. Together, these approaches allowed us to dissect how the E525K mutation perturbs key conformational equilibria in the myosin ATPase cycle.

### Salt sensitivity of actin-activated ATPase

**Figure 2:**
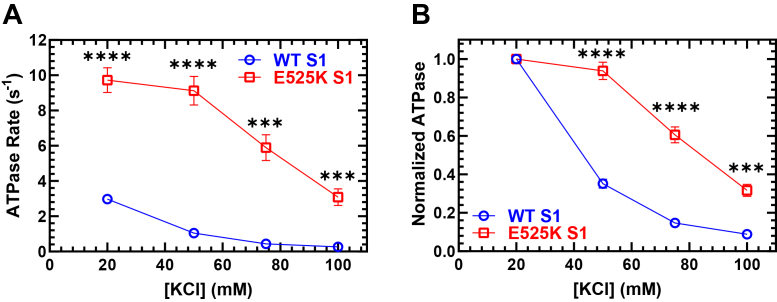
**Impact of salt concentration (KCl) on actin-activated ATPase activity.***A*, the actin-activated ATPase activity of purified M2β S1 WT and E525K was measured as a function of KCl concentration. *B*, normalized ATPase activity of M2β S1 WT and E525K under varying KCl concentrations. Data points represent the mean ± SD from three independent protein preparations. ∗∗∗∗*p* < 0.0001, ∗∗∗*p* < 0.001, unpaired *t* test for (*A*) and (*B*).

Our previous results demonstrated the E525K mutation in beta cardiac myosin subfragment 1 (S1) enhances actin-activated ATPase activity (Fig. 2*A* and (4)). Thus, we investigated the influence of this mutation on the so called “weak actin-binding” states, which are thought to be driven by electrostatic interactions during the initial stages of formation of the actomyosin complex. We examined the impact of salt concentration on the steady-state actin-activated ATPase reaction in WT and E525K S1 at a constant actin concentration (40 μM actin). We found that the E525K mutation was much less sensitive to increases in salt (KCl) concentration, which can be illustrated by comparing the relative decrease in ATPase activity observed in low salt (20 mM KCl) compared to higher salt (50, 75, and 100 mM KCl) conditions (Fig. 2, *A* and *B*). We observed a ∼60% reduction in ATPase when comparing the 20 and 50 mM KCl conditions in WT S1, while this only resulted in a ∼5% change in E525K S1 (Fig. 2*B*, Table S1). Furthermore, addition of 75 or 100 mM KCl resulted in an 80% and 90% reduction in ATPase for WT while only a 40% and 60% change in E525K (respectively) (Fig. 2*B*, Table S1). Our results agree with the hypothesis that E525K enhances actin attachment in the weak binding states making it less sensitive to changes in salt concentration.

### Salt sensitivity of in vitro motility

**Figure 3:**
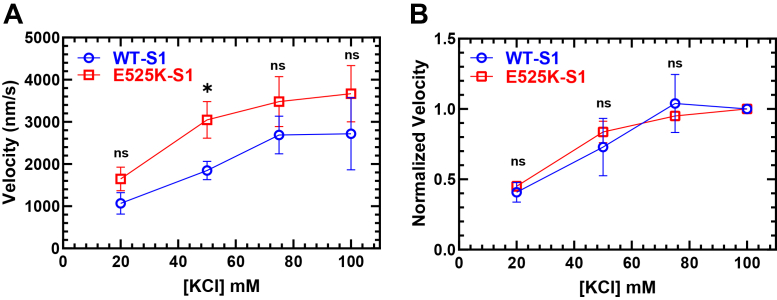
**Impact of salt concentration (KCl) on *in vitro* motility.***A*, the sliding velocity of actin filaments driven by purified M2β S1 WT and E525K mutant was measured as a function of KCl concentration. *B*, normalized sliding velocity of actin filaments under varying KCl concentrations. Data points represent the mean ± SD, calculated from an average of 75 actin filaments measured across three independent protein preparations. ∗*p* < 0.05, ns, not significant, unpaired *t* test for (*A*) and (*B*).

We investigated the impact of salt concentration on *in vitro* motility by varying the salt concentration in the same range that was examined in the ATPase experiments (Fig. 2). We observed faster *in vitro* actin gliding with E525K S1 in 50 mM KCl (Fig. 3*A*, Table S2), which is similar to what we reported previously (4). In both WT and E525K S1, the *in vitro* actin gliding velocity increases as a function of salt concentration in a hyperbolic manner and reached a maximum value at 75 mM KCl (Fig. 3*A*, Table S2). When the values were normalized to observe the relative change in sliding velocity as a function of salt concentration, we observed no difference between WT and E525K S1 (Fig. 3*B*, Table S2). Overall, the results indicate no difference in the salt concentration dependence of *in vitro* motility for WT and E525K S1, demonstrating that the enhanced actin-attachment in the weak binding states does not significantly impact *in vitro* actin gliding velocity.

### Impact of the E525K mutation on pyrene actin binding

**Figure 4:**
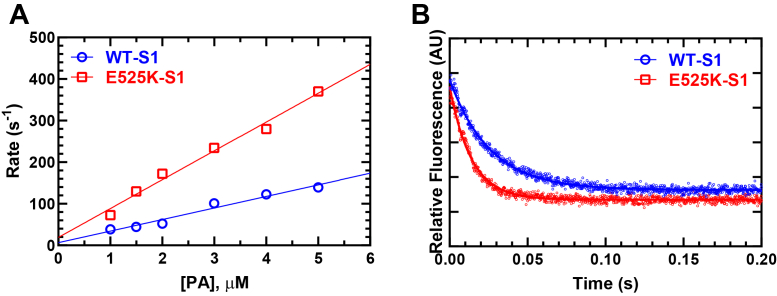
**Direct measurements of myosin binding to pyrene actin.***A*, the rate of WT and E525K M2β S1 binding to actin in the absence of nucleotide was monitored by pyrene actin quenching. Fluorescence transients of 5- to 10-fold excess pyrene actin mixed with M2β S1 were fit to a single exponential. The observed rates were linearly dependent on pyrene-actin concentration and the second-order binding constant was more than two-fold faster in E525K than in WT (74.9 ± 2.1 and 29.7 ± 1.1 μM^−1^ s^−1^, respectively). *B*, representative fluorescence transients of 1 μM pyrene actin mixed with 0.2 μM myosin (average of 3 transients) fit to a single exponential function (38.55 ± 0.32 and 72.48 ± 0.71, errors are SE of the fit).

Direct measurements of beta cardiac myosin-S1 binding to pyrene actin were performed in the stopped-flow by mixing pyrene-actin (5–10 fold excess) with either WT or E525K S1 in low salt conditions (20 mM KCl and 1 mM MgCl_2_) (Fig. 4, Table S3). The rate of fluorescence quenching, indicative of formation of the strong actomyosin complex, was fit to a single exponential function. The rate constants were linearly dependent on pyrene-actin concentration allowing us to determine the second-order binding constant, which was more than two-fold faster in E525K than in WT (74.9 ± 2.1 and 29.7 ± 1.1 μM^−1^ s^−1^, respectively). Overall, our results suggest the rate of actin attachment and transition into the strong actin-binding states is significantly accelerated by a single point mutation in the activation loop.

### Mg sensitivity of actin-activated ATPase

**Figure 5:**
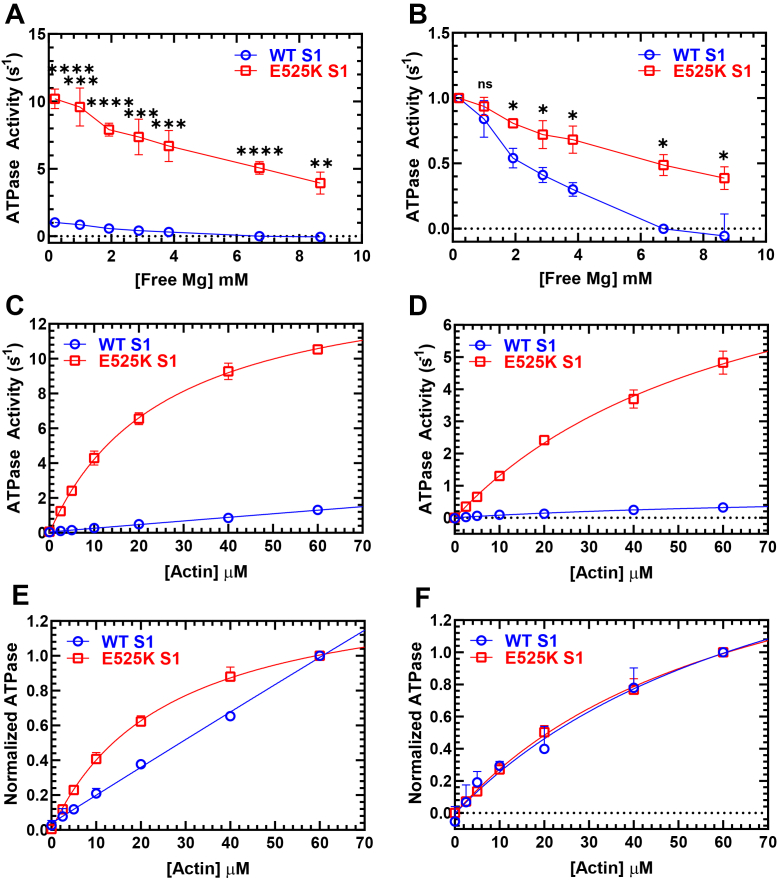
**Impact of free magnesium on actin-activated ATPase.***A*, the actin-activated ATPase activity of purified M2β S1 WT and E525K mutant was measured as a function of Mg^2+^ concentration. *B*, normalized ATPase activity of M2β S1 WT and E525K across varying free Mg concentrations. *C* and *D*, actin-activated ATPase activity of M2β S1 WT and E525K at 0.2 mM and 8.7 mM [free Mg], respectively, measured as a function of actin concentration. *E* and *F*, normalized ATPase activity of M2β S1 WT and E525K at 0.2 mM and 8.7 mM [free Mg], respectively, plotted as a function of actin concentration. Data points represent the mean ± SD from three independent protein preparations. ∗∗∗∗*p* < 0.0001, ∗∗∗*p* < 0.001, ∗∗*p* < 0.01, ∗*p* < 0.05, ns, not significant, unpaired *t* test for (*A*) and (*B*).

In our previous work, we observed that varying the MgCl_2_ concentration altered the ADP-release rate constant differently in E525K S1 compared to WT S1 (4), suggesting Mg coordination may be altered by the mutation. Thus, we studied the impact of free Mg concentration on ATPase activity, *in vitro* motility, and the ADP release rate constant under conditions where the ionic strength was held constant. We first examined actin-activated ATPase as a function of free Mg and held constant the total ionic strength by balancing the changes in Mg with the appropriate amount of KCl. As expected, based on our previous work examining the Mg dependence of actin-activated ATPase (9, 10), we observed decreases in the ATPase activity of both WT and E525K S1 as a function of free Mg (Fig. 5*A*, Table S4). The Mg-dependent decrease in actin-activated ATPase was more prominent in WT than in E525K S1 (4-fold in WT and 2-fold in E525K) (Fig. 5*B*, Table S4). To further investigate the actin-concentration at which ATPase is one-half maximal (K_ATPase_) and maximum ATPase activity (*k*_cat_) at low and high Mg concentrations, we performed the ATPase measurements as a function of actin concentration and fit the results to a Michaelis–Menten function to determine K_ATPase_ and *k*_cat_. We found that results with E525K S1 could be fit well to a hyperbolic function at low MgCl_2_, but WT S1 was linearly dependent on actin concentration preventing us from determining K_ATPase_ and *k*_cat_ (Fig. 5, *C* and *E*, Table S5). However, at high MgCl_2_, we were able to fit both the WT and E525K S1 ATPase results to a Michaelis–Menten function and thus determined K_ATPase_ and *k*_cat_. The *k*_cat_ was reduced 33% in E525K S1 in high compared to low free Mg conditions (15.2 ± 0.1 and 10.1 ± 1.0 s^−1^), while the K_ATPase_ was not significantly changed (Table S5). Overall, free Mg reduced the *k*_cat_ in E525K S1, indicating Mg may slow the rate-limiting step in the ATPase reaction.

### Mg sensitivity of in vitro motility

**Figure 6:**
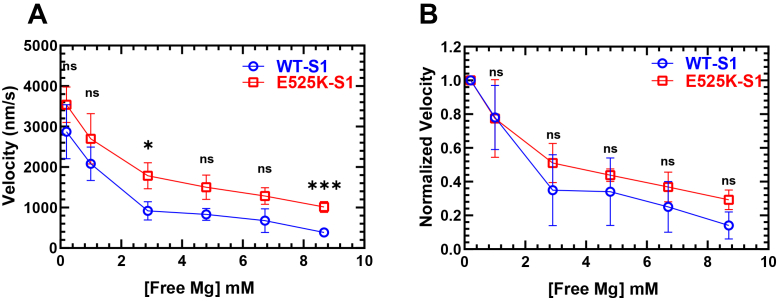
**Impact of free magnesium on *in vitro* motility.***A*, the sliding velocity of actin filaments driven by purified M2β S1 WT and E525K mutant was measured as a function of free Mg concentration. *B*, normalized sliding velocity of actin filaments under varying Mg^2+^ concentrations. Data points represent the mean ± SD, calculated from an average of 60 actin filaments measured across three independent protein preparations. ∗∗∗*p* < 0.001, ∗*p* < 0.05, ns, not significant, unpaired *t* test for (*A*) and (*B*).

We examined the Mg-dependence of *in vitro* motility under identical conditions that were used to measure ATPase. There was a dose-dependent decrease in actin gliding velocity for both WT and E525K S1 (Fig. 6*A*, Table S6). Representative actin filament movements at 0.2 and 8.7 mM free Mg are shown in Movies S1–S4. Examination of the normalized data revealed that Mg causes a similar reduction in the actin gliding velocity of WT S1 compared to E525K S1 (Fig. 6*B*, Table S6). Thus, actin gliding is similarly sensitive to Mg in WT compared to E525K S1.

### Impact of Mg on ADP release kinetics

**Figure 7:**
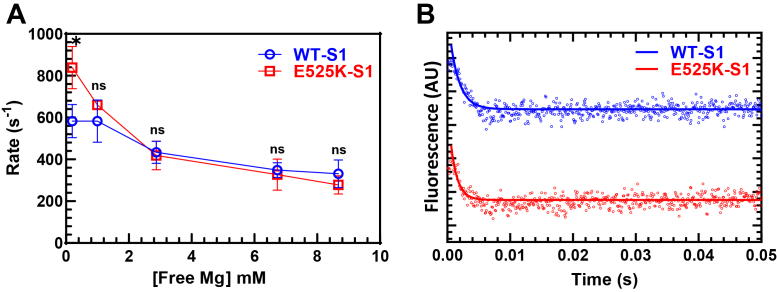
**Impact of free magnesium on the ADP release rate constant.** The ADP release rate constant was measured using mant-labeled ADP by mixing a complex of 1 μM M2β S1, 5 μM actin, and 20 μM mantADP with 2 mM ATP. *A*, the mantADP release rate constant was determined for WT and E525K mutant as a function of free Mg concentration. *B*, representative fluorescence transients of mantADP release from actomyosin (average of 3 transients) fit to a single exponential function. Data represents the mean ± SD from three independent experiments. ∗*p* < 0.05, ns, not significant, unpaired *t* test for (*A*).

Since the ADP-release rate constant is thought to be the rate limiting step in the *in vitro* actin gliding assay, we directly measured the release of mant-labeled ADP as a function of free Mg (Fig. 7, *A* and *B*, Table S7). In the stopped-flow apparatus, we mixed a complex of acto-S1. mant-ADP (5 μM actin, 1 μM S1, 20 μM mantADP) with saturating ATP (2 mM). At the lowest free Mg (196 μM), we observed a faster ADP-release rate constant in E525K compared to WT S1, while at all other Mg concentrations, we found very similar rate constants for the mutant and WT S1 (Fig. 7, *A* and *B*, Table S7). Thus, the faster ADP-release rate constant in E525K compared to WT at low free Mg indicates that E525K may alter Mg coordination in the active site.

### Impact of Mg on basal steady-state ATPase

**Figure 8:**
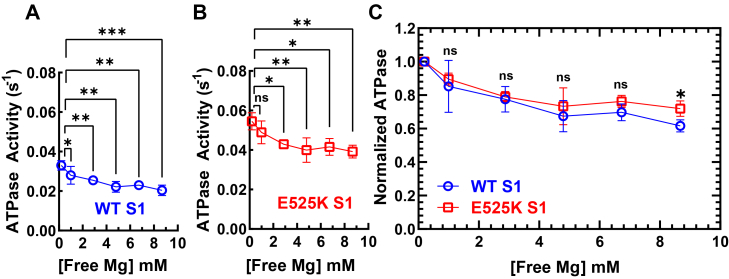
**Impact of free Mg on basal ATPase activity.** The ATPase activity in the absence of actin was measured as a function of time using the NADH coupled assay at varying Mg concentrations, while keeping the ionic strength constant for WT (*A*) and E525K (*B*). *C*, the data were replotted by normalizing the values to the lowest free Mg concentration, which demonstrated WT and E525K were similarly (∼25%) slowed by free Mg in the absence of actin. ∗∗∗*p* < 0.001, ∗∗*p* < 0.01, ∗*p* < 0.05, ns, not significant, one-way ANOVA for (*A*) and (*B*), unpaired *t* test for (*C*).

In the absence of actin (basal ATPase), the slowest step in the myosin ATPase reaction is thought to be the release of Pi from the active site (8, 12). Therefore, we examined the dose-dependence of free Mg-induced inhibition on the basal ATPase reaction (Fig. 8, *A* and *B*, Table S8). We found that both WT and E525K S1 have a similar dependence on free Mg (∼30–40% decrease at high, 8.7 mM, compared to low, 0.20 mM, Mg concentration), which suggests free Mg can also alter Pi release in the absence of actin.

### Molecular dynamics simulations

**Figure 9:**
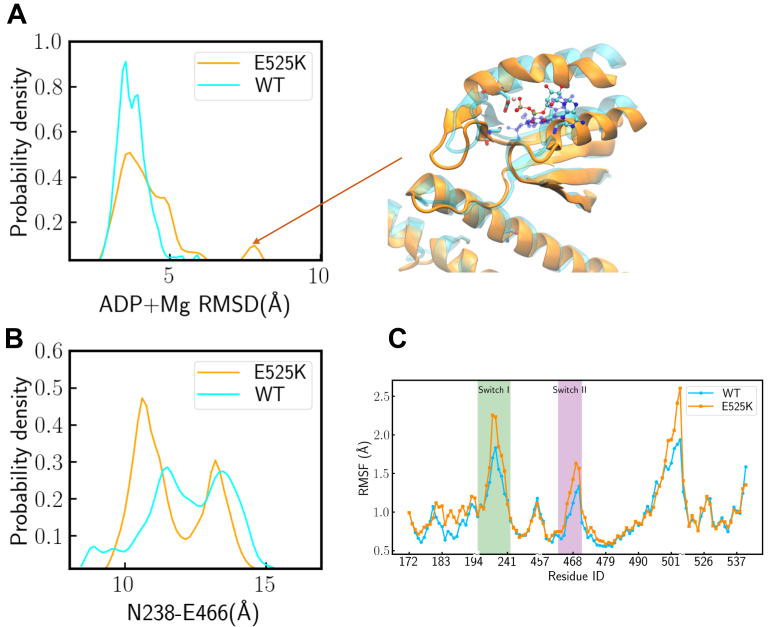
**Gaussian accelerated molecular dynamics simulations.***A*, the impact of E525K on the Mg-ADP binding in the active site, shown by the probability distributions of Mg-ADP RMSD. A second peak, representing a disengaged conformation of Mg-ADP, is observed in the mutant but not in WT. The *cyan* structure corresponds to the most probable WT state; the *orange* structure (aligned) corresponds to the disengaged state of E525K (*arrow*). *B*, the impact of E525K on the conserved switch I and switch II regions, demonstrated by probability distributions of the distance between N238 (switch I) and E466 (switch II). The first peak includes the ADP disengaged state in (*A*), in which switch I moved away from Mg-ADP. *C*, root-mean-square fluctuation (RMSF) of the switch I and switch II regions is also enhanced by the E525K mutation.

In order to examine the impact of E525K on Mg-ADP binding and allosteric effects, we performed Gaussian-accelerated molecular dynamics (GaMD) simulations on the actomyosin.ADP state structure with and without the mutation. The simulations revealed that E525K shows a small probability of a disengaged Mg-ADP state, whereas this state is not observed in the WT simulations (Fig. 9*A*). Additionally, the E525K mutant exhibits a shift in its main peak toward larger root-mean square deviation values. Root-mean-square fluctuation analysis shows the dynamics of key motifs are enhanced by the E525K mutation in the ADP-bound state (Fig. 9*C*). E525K also favors a closer distance between N238 (switch I) and E466 (switch II), as indicated by the first peak in the distribution (Fig. 9*B*). This higher peak of E525K includes the disengaged Mg-ADP state, in which switch I moves away from Mg-ADP, allowing the nucleotide to shift out of the pocket. The other regions of myosin were also examined, excluding surface loops, but we did not observe major changes in the root-mean-square fluctuation (Fig. S1).

## Discussion

The current study provides strong evidence that the activation loop plays a key role in the so called “weak-binding” states and/or the transition from “weak” to “strong” actin binding. The activation loop may be crucial for the docking of the lower 50 kDa domain onto the negatively charged N terminus of actin, which has been proposed to be one of the first steps that occurs during the formation of the actomyosin complex and involves both electrostatic and hydrophobic interactions (3). Increasing the positive charge on the activation loop, such as with the E525K mutation, appears to have a dramatic impact on actin attachment and thus actin-activation of the ATPase reaction. Our Mg-dependent studies also provide strong evidence that there is allosteric communication between the activation loop and active site. Our work provides specific details about how free Mg can regulate human beta-cardiac myosin ATPase and motor activity.

### Mechanism of actin-activation: Role of the initial electrostatic interaction with actin and the activation loop

Previous work has highlighted the activation loop as a key region for enhancing actin-activation (6). It was demonstrated that mutation of a highly conserved lysine in the activation loop, K526 in cardiac myosin, to a negatively charged or neutral residue reduced the maximum actin-activated ATPase rate but did not alter actin filament sliding in class II and V myosins (6). They hypothesized that docking of the activation loop on actin was required to allosterically trigger lever arm rotation and thus efficient ATPase cycles. Since the critical lysine (K526) is adjacent to E525 that forms a salt bridge to K484 on the relay helix, we suggest that the 484 to 525 salt bridge is involved in allosterically coordinating actin binding, lever arm rotation, and phosphate release. Introducing the E525K mutation results in two adjacent lysines that may both interact with the N terminus of actin during the weak binding states, which dramatically accelerates the transition into strong actin binding. Interestingly, abolishing the 484 to 525 salt bridge does not diminish motor function and indeed E525K enhances lever arm rotation, force generation, and phosphate release (4). Thus, keeping the 484 to 525 salt-bridge intact is not as important as docking the activation loop onto actin.

Loop 2 is another loop that is thought to form electrostatic interactions with actin to promote the initial interaction with actin in the weak binding states (13, 14, 15, 16, 17, 18, 19, 20). This loop is highly charged and variable in different myosin isoforms. There are some reports that suggest loop 2 is simply an electrostatic tether (16) while others suggest it is important in triggering actin-activated phosphate release (18). The helix-loop-helix motif and loop 3, located on the lower 50 kDa domain, are known to be part of the initial actin-binding interface (3). In the upper 50 kDa domain, loop 4 and the cardiomyopathy loop are known to interact with actin but proposed to play a role mainly after actin binding cleft closure and strong actin binding (3). Thus, a common theme of the actomyosin interaction is that electrostatic mechanisms dominate the initial attachment, while in some cases, they may also trigger a conformational change that is associated with formation of the strong actomyosin complex and acceleration of product release as well as lever arm rotation.

We hypothesize the following sequence of events based on our current work and many other published studies (3, 15). The positively charged loop 2 functions as an electrostatic tether by interacting with the negatively charged N terminus of actin. The activation loop then also forms electrostatic interactions with the N terminus of actin, which allows for the lower 50 kDa domain to properly dock onto actin. The lower 50 kDa helix-loop-helix and loop 3 motifs are crucial for this docking, which is promoted by both ionic and hydrophobic interactions. The transition to a strongly bound actomyosin complex is associated with several important conformational changes in myosin, including opening the phosphate release tunnel to allow phosphate release, closure of the actin-binding cleft which allows binding of the upper 50 kDa domain to actin, and a swing of the lever arm form the pre- to post-power stroke conformation.

An alternative hypothesis is that E525K indirectly alters actomyosin attachment by altering the conformation of loop 2. This was proposed by the Regnier group based on molecular dynamics simulations, which demonstrated that the E525K mutation causes loop 2 to favor a more extended conformation that can freely form electrostatic interactions with actin (21). They point out that the E525K mutation disrupts interactions with amino acids that can influence the conformation of a helix at the base of loop 2 and thus could impact the conformation and or dynamics of loop 2. Further studies may be necessary to determine if the E525K mutation enhances actin attachment by directly enhancing the interaction of the activation loop with actin or indirectly altering the conformation of loop 2 or both.

### Mg sensitivity and allosteric communication between the activation loop and active site

Previous work demonstrated that the activation loop may play a critical role in triggering the release of phosphate and lever arm rotation (4, 6). Our recent work demonstrated directly that the E525K mutation can enhance the phosphate release rate constant as well as the rate constant associated with lever arm rotation during the power stroke (4). Our molecular dynamics simulations supported our hypothesis that E525 can form a salt bridge with K484 on the relay helix further establishing the allosteric connection between the activation loop and the active site (4). The allosteric mechanism coordinates structural changes in the lever arm, actin-binding region, and active site associated with product release (4). Interestingly, we found that the E525K mutation increases the basal ATPase activity without actin, suggesting the activation loop is allosterically linked to the active site even in the absence of actin (4). Therefore, we further explored the allosteric link in the current study by examining the impact of the mutation on Mg-sensitivity.

Mg is coordinated in the active site by conserved amino acids, threonine 185 in the P-loop and serine 242 in switch I, as well as indirectly through aspartate 461 which coordinates an interacting water molecule (Fig. 1) (7, 22). Mg is also coordinated by oxygen atoms in the beta- and gamma-phosphate of ATP. After the release of phosphate, the loss of the gamma phosphate is thought to alter Mg coordination in the active site considerably. Previous work has demonstrated that increases in free Mg can slow the ADP release rate constant, which is thought to limit detachment from actin and the rate of actin filament gliding in the motility assay (9, 10, 11). The mechanism first proposed by Rosenfeld and Sweeney (11) suggests that Mg can dissociate from the active site prior to ADP dissociation which accelerates nucleotide release because ADP has a weaker affinity for the active site than Mg-ADP. In the current study, we also found that free Mg can slow the ADP release rate constant and *in vitro* actin gliding in the motility assay, which is consistent with previous reports (9). The E525K mutation has an accelerated ADP release rate constant at low free Mg concentrations compared to WT. Thus, the E525K mutation may alter the conformation of the active site such that Mg coordination is disrupted and thus at lower free Mg concentrations, ADP release is more likely to occur without Mg bound, accelerating the process. However, we did not observe major changes in the degree of Mg sensitivity in the *in vitro* motility assay between WT and E525K. As we noted in our previous work, E525K does display faster *in vitro* motility than WT despite having a similar ADP-release rate constant at higher Mg concentrations (4). Overall, our results suggest that the ADP-release rate constant, and hence detachment from actin, may not be the only factor that mediates the unloaded actin filament gliding in the motility assay. Indeed, attachment limited mechanisms have been reported and could play a role (23, 24). For example, in low free Mg conditions, the very rapid rate of ADP release in E525K may exceed that of actin attachment and thus favor an attachment limited regime.

Our GaMD results also support the conclusion that there is an allosteric pathway from the activation loop to the active site (Fig. 9). We found that Mg-ADP coordination is altered in actomyosin E525K, which results in larger fluctuations in the motifs that bind Mg-ADP in the active site. The mutant was shown to populate an alternative conformation of the active site in which the Mg-ADP is disengaged and thus would likely dissociate more rapidly. When examining the conserved switch I and switch II regions, we found that there is an enhanced probability of adopting a conformation where switch I moves away from Mg-ADP and thus is more likely to dissociate. However, we did not observe conformations in which Mg moved away from ADP in the active site as a result of the mutation.

Free Mg was also demonstrated to slow steady-state actin-activated ATPase, which is thought to be limited by attachment to actin and actin-activated phosphate release in myosin II (25, 26). However, the mechanism of altering phosphate release with free Mg is unclear because Mg is not thought to exchange in the active site until after phosphate release, since it is highly coordinated with the gamma-phosphate (22). In the current study, we did find that free Mg alters the maximum rate of actin-activated ATPase in the E525K mutant. It is possible that Mg alters actin binding which would indirectly impact the actin-activated ATPase rate. In a previous study with myosin V, we performed direct measurements of phosphate release and found that the actin affinity was reduced by free Mg but the maximum rate of phosphate release was unchanged (10). The results with myosin V suggest free Mg alters a conformational change that alters actin affinity, which is also a possibility in the current study of beta-cardiac myosin. In addition, we measured the basal ATPase activity as a function of free Mg, which is thought to be limited by phosphate release. We found a dose-dependent decrease in basal ATPase as a function of free Mg, which suggests a conformation change, not associated with actin binding, is sensitive to free Mg. Thus, novel allosteric free Mg-binding sites, other than the active site, may be present on myosin. A previous study demonstrated non-active site associated phosphate binding sites in myosin (27), and thus allosteric sites for free Mg are also possible.

### Physiological implications of free Mg-dependent regulation of cardiac myosin

The intracellular concentration of free Mg in cardiac muscle is 1 to 2 mM and thus variation in free Mg may not be expected to alter contractility during normal physiological conditions (28, 29). However, the free Mg may increase during ischemia reperfusion which is known to be associated with periods of hypercontractility (28, 29). In addition, changing the ATP levels or ATP/ADP ratio can alter free Mg significantly and thus alter contractility. Indeed, we and others have demonstrated mitochondria dysfunction in different forms of heart failure (30, 31), which could lead to a reduction in ATP production and thus increase free Mg. Therefore, our results which shed light on the molecular mechanisms of Mg-associated regulation of cardiac myosin function may provide insight into contractility dysfunction in different physiological and disease conditions.

## Conclusions

The current study provides key insight into several critical aspects of myosin mechanochemistry. First, we established that the activation loop’s interaction with actin can mediate the initial attachment to actin and transition into the strong actin-binding conformation. The activation loop is also involved in triggering actin-activation of phosphate release and lever arm rotation, as demonstrated in our previous work (4). Second, we further established the allosteric connection between the activation loop and the active site based on our results that demonstrate Mg-dependence of ADP and phosphate release are altered by the E525K mutation. Our results also suggest free Mg can alter phosphate release in cardiac myosin perhaps through unexplored mechanisms. Overall, we find that the activation loop, which directly interacts with actin and can influence the conformation of the relay helix, is a crucial factor in mediating attachment to actin and actin-activation of lever arm rotation and phosphate release.

## Experimental procedures

### Reagents

All experiments were performed in Mops buffer (10 mM Mops, pH 7.0, 20–150 mM KCl, 1–10 mM MgCl_2_, 1 mM EGTA, 1 mM DTT) at 25 °C. Additional KCl was added to achieve the target ionic strength. For Mg-dependence experiments, MgCl_2_ concentration was varied and KCl concentration was adjusted to balance total ionic strength. ATP was prepared from powder (Millipore-Sigma), and its concentration was determined by absorbance at 259 nm using an extinction coefficient of ε = 15,400 M^−1^ cm^−1^. 2'/3′-O-(N-Methyl-anthraniloyl)-adenosine-5′-diphosphate (mant-ADP) was purchased from Jena Biosciences.

### Protein expression and purification

Human β-cardiac myosin subfragment 1 (M2β S1) constructs containing a C-terminal Avi and FLAG tag were cloned into the pDual shuttle vector as previously described (4). The E525K mutation was introduced into the constructs using the Quikchange site-directed mutagenesis (Agilent). Recombinant adenovirus was generated using Vector Biolabs and amplified in Ad293 cells. C2C12 mouse skeletal muscle cells were infected at ∼90% confluency with virus (4 × 10^7^ pfu/ml) in differentiation medium (4, 32). Cells were harvested 10 to 12 days postinfection, and myosin proteins were purified using FLAG affinity chromatography (4, 32). The recombinant β-cardiac myosin contained endogenous mouse light chains and the purity was assessed by SDS-PAGE (4, 32).

Rabbit skeletal muscle actin was purified from acetone powder (Pel-Freez Biologicals) (33) and polymerized to F-actin *via* dialysis into experimental buffer. F-actin was labeled with pyrene iodoacetamide to directly examine myosin binding to actin (34). For *in vitro* motility, Alexa Fluor 555–phalloidin was used to label actin filaments.

### Steady-state ATPase assays

Steady-state actin-activated ATPase activity was measured using an NADH-coupled assay as described in previous studies (4, 35, 36). Final reaction mixtures included 0.1 μM myosin, varying actin concentrations (5–60 μM), 2 mM ATP, 0.2 mM NADH, 0.46 mM phosphoenolpyruvate, 46 units/ml pyruvate kinase, and 11 units/ml lactate dehydrogenase. Absorbance at 340 nm was monitored for 200 s and converted to ADP/sec using an ADP standard curve. The ATPase rate was plot as a function of actin concentration and the data fit to a hyperbolic function to determine the maximum ATPase rate (*k*_cat_) and actin concentration as which ATPase is one-half maximal (*K*_ATPase_).

### *In vitro* motility assay

Flow chambers were assembled using coverslips coated with 0.2% nitrocellulose and anti-GFP antibody to immobilize GFP-tagged M2β S1 (4). Chambers were blocked with 1 mg/ml BSA, followed by the addition of 0.5 μM myosin, sheared unlabeled actin, and ATP to block inactive heads. Alexa-555–phalloidin-labeled actin filaments were introduced with motility buffer containing 2 mM ATP, an oxygen scavenger system, and methylcellulose. Actin gliding was recorded using a Leica DMi8 fluorescence microscope and analyzed using ImageJ (MTrackJ plugin) (4, 37).

### Transient kinetic experiments

Mant-labeled ADP (Jena Bioscience) was used to monitor nucleotide binding and release in the Applied Photophysics Stopped-Flow apparatus equipped with an excitation monochrometer (band pass) and emission filters (4, 32). Fluorescence was monitored (290 nm excitation, 395 nm long-pass emission filter), and data were fit to exponential functions to determine rate constants. Binding to pyrene actin was monitoring by following the rate of pyrene-actin quenching (365 nm excitation, 395 nm long-pass emission filter) with 5- to 10-fold excess pyrene actin relative to myosin concentration.

### Statistics

Data are reported as mean ± SD from a minimum of three independent protein preparations. Curve fits and statistical comparisons (Student’s t-tests or one-way ANOVA) were performed using GraphPad Prism. *p* values < 0.05 were considered statistically significant.

### Gaussian-accelerated molecular dynamics simulations

The cardiac actin–myosin complex (ADP state, PDB ID 8EFH) (38) was used as the starting structure for the MD simulations. Hydrogen atoms were added using MolProbity (39), and E525K mutation was introduced using PyMOL. Both the WT and E525K models were solvated in a water box ionized to 150 mM NaCl. All the simulations were performed with the GPU-enabled Amber24 package (40) using the ff19SB force field (41). Each system was energy-minimized for 2000 steps with harmonic restraint (25 kcal mol^−1^ Å^−2^) applied to protein and ligand heavy atoms. For equilibration, we performed an initial 1 ns NVT run followed by seven consecutive NPT runs of 500 ps each, during which the restraint force constant was gradually reduced to 0.1 kcal mol^−1^ Å^−2^. The temperature was maintained at 300K by Langevin dynamics with a friction coefficient of 1 ps^−1^. GaMD was employed to enhance the sampling of protein conformational dynamics (42, 43). First an 8-ns of conventional MD stage was used to collect statistics for determining the initial GaMD acceleration parameters. Then a 100-ns GaMD stage was conducted under NPT conditions at 1 bar and 300 K. Both total-potential energy boost and dihedral energy boost were applied, with the SD of each boost limited to 6 kcal/mol to ensure reliable reweighting. Ten independent replicas were performed for each of the WT and mutant systems, yielding a total accumulated GaMD sampling time of 2 μs. Free energy profiles were computed using the reweighting procedure described in *Ma et al.* (43).

## Data availability

Data will be shared upon request to Christopher M. Yengo (cmy11@psu.edu)

## Supporting information

This article contains supporting information.

## Conflicts of interest

The authors declare that they have no conflicts of interest with the contents of this article.

## References

[1] Sweeney H.L., Holzbaur E.L.F. (2018). Motor proteins. Cold Spring Harb. Perspect. Biol..

[2] Preller M., Manstein D.J. (2013). Myosin structure, allostery, and mechano-chemistry. Structure.

[3] Robert-Paganin J., Pylypenko O., Kikuti C., Sweeney H.L., Houdusse A. (2020). Force generation by myosin motors: a structural perspective. Chem. Rev..

[4] Bodt S.M.L., Ge J., Ma W., Rasicci D.V., Desetty R., McCammon J.A. (2024). Dilated cardiomyopathy mutation in beta-cardiac myosin enhances actin activation of the power stroke and phosphate release. PNAS Nexus.

[5] Rasicci D.V., Tiwari P., Bodt S.M.L., Desetty R., Sadler F.R., Sivaramakrishnan S. (2022). Dilated cardiomyopathy mutation E525K in human beta-cardiac myosin stabilizes the interacting-heads motif and super-relaxed state of myosin. Elife.

[6] Varkuti B.H., Yang Z., Kintses B., Erdelyi P., Bardos-Nagy I., Kovacs A.L. (2012). A novel actin binding site of myosin required for effective muscle contraction. Nat. Struct. Mol. Biol..

[7] Kozlova M.I., Shalaeva D.N., Dibrova D.V., Mulkidjanian A.Y. (2022). Common mechanism of activated catalysis in P-loop fold nucleoside triphosphatases-united in diversity. Biomolecules.

[8] Bagshaw C.R., Eccleston J.F., Eckstein F., Goody R.S., Gutfreund H., Trentham D.R. (1974). The magnesium ion-dependent adenosine triphosphatase of myosin. Two-step processes of adenosine triphosphate association and adenosine diphosphate dissociation. Biochem. J..

[9] Swenson A.M., Trivedi D.V., Rauscher A.A., Wang Y., Takagi Y., Palmer B.M. (2014). Magnesium modulates actin binding and ADP release in myosin motors. J. Biol. Chem..

[10] Trivedi D.V., Muretta J.M., Swenson A.M., Thomas D.D., Yengo C.M. (2013). Magnesium impacts myosin V motor activity by altering key conformational changes in the mechanochemical cycle. Biochemistry.

[11] Rosenfeld S.S., Houdusse A., Sweeney H.L. (2005). Magnesium regulates ADP dissociation from myosin V. J. Biol. Chem..

[12] Bagshaw C.R., Trentham D.R. (1974). The characterization of myosin-product complexes and of product-release steps during the magnesium ion-dependent adenosine triphosphatase reaction. Biochem. J..

[13] Onishi H., Mikhailenko S.V., Morales M.F. (2006). Toward understanding actin activation of myosin ATPase: the role of myosin surface loops. Proc. Natl. Acad. Sci. U. S. A..

[14] Rovner A.S. (1998). A long, weakly charged actin-binding loop is required for phosphorylation-dependent regulation of smooth muscle myosin. J. Biol. Chem..

[15] Klebl DP, McMillan SN, Risi C, Forgacs E, Virok B, Atherton JL (2025). Swinging lever mechanism of myosin directly shown by time-resolved cryo-EM. Nature.

[16] Yengo C.M., Sweeney H.L. (2004). Functional role of loop 2 in myosin V. Biochemistry.

[17] Furch M., Geeves M.A., Manstein D.J. (1998). Modulation of actin affinity and actomyosin adenosine triphosphatase by charge changes in the myosin motor domain. Biochemistry.

[18] Joel P.B., Trybus K.M., Sweeney H.L. (2001). Two conserved lysines at the 50/20-kDa junction of myosin are necessary for triggering actin activation. J. Biol. Chem..

[19] Knetsch M.L., Uyeda T.Q., Manstein D.J. (1999). Disturbed communication between actin- and nucleotide-binding sites in a myosin II with truncated 50/20-kDa junction. J. Biol. Chem..

[20] Kojima S., Konishi K., Katoh K., Fujiwara K., Martinez H.M., Morales M.F. (2001). Functional roles of ionic and hydrophobic surface loops in smooth muscle myosin: their interactions with actin. Biochemistry.

[21] Kalen Z., Roberson M.C.C., Davis J.M., Regnier M. (2025). The role of loop 2 conformations in actomyosin complex formation and cardiomyopathy caused by the mutations E252K and V606M. Biophysical J..

[22] Smith C.A., Rayment I. (1996). X-ray structure of the magnesium(II).ADP.vanadate complex of the dictyostelium discoideum myosin motor domain to 1.9 a resolution. Biochemistry.

[23] Brizendine R.K., Alcala D.B., Carter M.S., Haldeman B.D., Facemyer K.C., Baker J.E. (2015). Velocities of unloaded muscle filaments are not limited by drag forces imposed by myosin cross-bridges. Proc. Natl. Acad. Sci. U. S. A..

[24] Stewart T.J., Murthy V., Dugan S.P., Baker J.E. (2021). Velocity of myosin-based actin sliding depends on attachment and detachment kinetics and reaches a maximum when myosin-binding sites on actin saturate. J. Biol. Chem..

[25] Lymn R.W., Taylor E.W. (1971). Mechanism of adenosine triphosphate hydrolysis by actomyosin. Biochemistry.

[26] Geeves M.A. (2016). Review: the ATPase mechanism of myosin and actomyosin. Biopolymers.

[27] Moretto L., Usaj M., Matusovsky O., Rassier D.E., Friedman R., Mansson A. (2022). Multistep orthophosphate release tunes actomyosin energy transduction. Nat. Commun..

[28] Headrick J.P., Willis R.J. (1991). Cytosolic free magnesium in stimulated, hypoxic, and underperfused rat heart. J. Mol. Cell Cardiol..

[29] Murphy E., Steenbergen C., Levy L.A., Raju B., London R.E. (1989). Cytosolic free magnesium levels in ischemic rat heart. J. Biol. Chem..

[30] Rasicci D.V., Ge J., Chen A.P., Wood N.B., Bodt S.M.L., Toro A.L. (2025). Early-stage alcoholic cardiomyopathy highlighted by metabolic remodeling, oxidative stress, and cardiac myosin dysfunction in Male rats. Int. J. Mol. Sci..

[31] Li A., Shami G.J., Griffiths L., Lal S., Irving H., Braet F. (2023). Giant mitochondria in cardiomyocytes: cellular architecture in health and disease. Basic Res. Cardiol..

[32] Swenson A.M., Tang W., Blair C.A., Fetrow C.M., Unrath W.C., Previs M.J. (2017). Omecamtiv mecarbil enhances the duty ratio of human beta-Cardiac myosin resulting in increased calcium sensitivity and slowed force development in cardiac muscle. J. Biol. Chem..

[33] Pardee J.D., Spudich J.A. (1982). Purification of muscle actin. Methods Enzymol..

[34] Pollard T.D. (1984). Polymerization of ADP-actin. J. Cell Biol..

[35] Duno-Miranda S., Nelson S.R., Rasicci D.V., Bodt S.M.L., Cirilo J.A., Vang D. (2024). Tail length and E525K dilated cardiomyopathy mutant alter human beta-cardiac myosin super-relaxed state. J. Gen. Physiol..

[36] Tang W., Unrath W.C., Desetty R., Yengo C.M. (2019). Dilated cardiomyopathy mutation in the converter domain of human cardiac myosin alters motor activity and response to omecamtiv mecarbil. J. Biol. Chem..

[37] Meijering E., Dzyubachyk O., Smal I. (2012). Methods for cell and particle tracking. Methods Enzymol..

[38] Doran M.H., Rynkiewicz M.J., Rasicci D., Bodt S.M.L., Barry M.E., Bullitt E. (2023). Conformational changes linked to ADP release from human cardiac myosin bound to actin-tropomyosin. J. Gen. Physiol..

[39] Williams C.J., Headd J.J., Moriarty N.W., Prisant M.G., Videau L.L., Deis L.N. (2018). MolProbity: more and better reference data for improved all-atom structure validation. Protein Sci..

[40] Salomon-Ferrer R., Case D.A., Walker R.C. (2013). An overview of the amber biomolecular simulation package. WIREs Comput. Mol. Sci..

[41] Tian C., Kasavajhala K., Belfon K.A.A., Raguette L., Huang H., Migues A.N. (2020). ff19SB: Amino-acid-specific protein backbone parameters trained against Quantum Mechanics Energy surfaces in solution. J. Chem. Theory Comput..

[42] Miao Y., Feher V.A., McCammon J.A. (2015). Gaussian accelerated molecular dynamics: unconstrained enhanced sampling and free energy calculation. J. Chem. Theory Comput..

[43] Ma W., You S., Regnier M., McCammon J.A. (2023). Integrating comparative modeling and accelerated simulations reveals conformational and energetic basis of actomyosin force generation. Proc. Natl. Acad. Sci. U. S. A..

