# Supplementary material for: Impact of salt concentration and free magnesium on human beta-cardiac myosin reveal important details about the conserved mechanochemical mechanism
Source: J Biol Chem. 2026 May 6;302(6):113114. doi: 10.1016/j.jbc.2026.113114 (PMC13264367; doi:10.1016/j.jbc.2026.113114)
Supplement: Supplementary Data [file mmc5.docx]

**Supporting Information**

**Impact of salt concentration and free magnesium on human beta-cardiac myosin reveal important details about the conserved mechanochemical mechanism**

Jinghua Ge^1^, Michael R. Ebert^1^, Skylar M.L. Bodt^1^, Satyabrata Majumder^2^, Wen Ma^2^, and Christopher M. Yengo*^1^

^1^Department of Cell and Biological Systems, Penn State College of Medicine, Hershey, PA 17033

^2^Department of Physics, University of Vermont, Burlington, VT 05405

*corresponding author. [cmy11@psu.edu](mailto:cmy11@psu.edu)

Supplemental Movies #1-4

Table S1. Impact of ionic strength on actomyosin ATPase.

Table S2. Impact of ionic strength on in vitro motility.

Table S3. Pyrene actin binding in the absence of nucleotide.

Table S4. Impact of Mg on actin-activated ATPase.

Table S5. Actin-activated ATPase as a function of free Mg.

Table S6. Impact of Mg on in vitro motility.

Table S7. Impact of Mg on ADP release.

Table S8. Impact of Mg on basal ATPase.

Figure S1. RMSF profiles of the myosin motor domain from WT and E525K GaMD simulations.

**Supplemental Movies #1-4.** Representative movies of in vitro motility for WT and E525K beta-cardiac myosin S1 in low (1 mM) and high (10 mM) MgCl_2_ conditions. Each movie is labeled with the construct, MgCl_2_ concentration, and contains a 10 µm scale bar.

**Table S1.** Impact of ionic strength on actomyosin ATPase (40 µM actin and 0.1 µM myosin). Errors are SD (N=3).

| KCl  concentration | WT  ATPase (s^-1^) | E525K  ATPase (s^-1^) | P value  WT vs E525K | WT  ATPase (s^-1^)  (Relative to 20 mM KCl) | E525K  ATPase (s^-1^)  (Relative to 20 mM KCl) | P value  WT vs E525K  (Relative to 20 mM KCl) |
| --- | --- | --- | --- | --- | --- | --- |
| 20 mM | 2.97±0.13 | 9.73±0.70 | <0.0001 | 1 | 1 | - |
| 50 mM | 1.05±0.11 | 9.13±0.81 | <0.0001 | 0.35±0.02 | 0.94±0.04 | <0.0001 |
| 75 mM | 0.44±0.06 | 5.90±0.74 | 0.0002 | 0.15±0.01 | 0.61±0.04 | <0.0001 |
| 100 mM | 0.26±0.07 | 3.09±0.48 | 0.0005 | 0.09±0.02 | 0.32±0.03 | 0.0004 |

**Table S2.** Impact of ionic strength on in vitro motility. Errors are SD (N=3).

| KCl concentration | WT  IVM (nm/s) | E525K  IVM (nm/s) | P value  WT vs E525K | WT  IVM (nm/s)  (Relative to 100 mM KCl) | E525K  IVM (nm/s)  (Relative to 100 mM KCl) | P value  WT vs E525K  (Relative to 100 mM KCl) |
| --- | --- | --- | --- | --- | --- | --- |
| 20 mM | 1069±257 | 1648±321 | 0.0718 | 0.41±0.07 | 0.45±0.04 | 0.3814 |
| 50 mM | 1850±188 | 3046±515 | 0.0195 | 0.73±0.20 | 0.84±0.08 | 0.4290 |
| 75 mM | 2690±490 | 3480±679 | 0.1773 | 1.04±0.21 | 0.95±0.02 | 0.5166 |
| 100 mM | 2720±1019 | 3667±770 | 0.2680 | 1 | 1 | - |

**Table S3.** Pyrene actin binding in the absence of nucleotide. Errors are SE of the fit.

| Construct | Rate of attachment to actin  (µM^-1^∙s^-1^) |
| --- | --- |
| WT | 29.7±1.1 |
| E525K | 74.9±2.1 |
| P value | <0.0001 |

**Table S4.** Impact of Mg on actin-activated ATPase (40 µM actin and 0.1 µM myosin). Errors are SD (N=3).

| Free Mg concentration | WT  ATPase (s^-1^) | E525K  ATPase (s^-1^) | P value  WT vs E525K | WT  ATPase  (Relative to 0.2 mM Mg) | E525K  ATPase  (Relative to 0.2 mM Mg) | P value  WT vs E525K  (Relative to 0.2 mM Mg) |
| --- | --- | --- | --- | --- | --- | --- |
| 0.20 mM | 1.02±0.08 | 10.18±0.72 | <0.0001 | 1 | 1 | - |
| 1.0 mM | 0.85±0.08 | 9.58±1.41 | 0.0004 | 0.84±0.14 | 0.94±0.07 | 0.3430 |
| 1.9 mM | 0.56±0.03 | 7.90±0.49 | 0.0001 | 0.54±0.07 | 0.81±0.03 | 0.0402 |
| 2.9 mM | 0.42±0.03 | 7.36±1.32 | 0.0008 | 0.41±0.06 | 0.72±0.11 | 0.0112 |
| 3.8 mM | 0.31±0.03 | 6.68±1.16 | 0.0007 | 0.30±0.05 | 0.68±0.10 | 0.0451 |
| 6.7 mM | -0.00±0.03 | 5.06±0.47 | 0.0001 | 0.00±0.02 | 0.49±0.08 | 0.0150 |
| 8.7 mM | -0.06±0.16 | 3.93±0.82 | 0.0011 | -0.05±0.17 | 0.39±0.09 | 0.0157 |

**Table S5.** Actin-activated ATPase as a function of free Mg. Errors are SE of the fit except ATPase at 60 µM Actin, which is SD (N=3).

| Free Mg concentration | WT  *k*_cat_ (s^-1^) | E525K  *k*_cat_ (s^-1^) | P value  *k*_cat_ | WT  ATPase (s^-1^) (60 µM Actin) | E525K  ATPase (s^-1^) (60 µM Actin) | P value  ATPase (60 µM Actin) | WT  K_ATPase_(µM) | E525K  K_ATPase_(µM) | P value  K_ATPase_ |
| --- | --- | --- | --- | --- | --- | --- | --- | --- | --- |
| 0.20 mM | ND | 15.2±0.1 | - | 1.3±0.1 | 10.5±0.2 | 0.0001 | ND | 25.9±2.2 | - |
| 8.7 mM | 0.8±0.3 | 10.1±1.0 | 0.0009 | 0.3±0.1 | 4.8±0.4 | 0.0004 | 91.3±61.0 | 66.7±12.3 | 0.7128 |

**Table S6.** Impact of Mg on in vitro motility. Errors are SD (N=3).

| Free Mg concentration | WT  IVM  (nm∙s^-1^) | E525K  IVM  (nm∙s^-1^) | P value  WT vs E525K | WT  IVM  (Relative to 0.2 mM Mg) | E525K  IVM  (Relative to 0.2 mM Mg) | P value  WT vs E525K  (Relative to 0.2 mM Mg) |
| --- | --- | --- | --- | --- | --- | --- |
| 0.20 mM | 2965±854 | 3541±486 | 0.3675 | 1.00±0.29 | 1.00±0.14 | - |
| 1.0 mM | 2249±688 | 2699±731 | 0.4805 | 0.76±0.23 | 0.76±0.21 | 0.9891 |
| 2.9 mM | 920±234 | 1785±358 | 0.0250 | 0.31±0.08 | 0.50±0.10 | 0.2917 |
| 4.8 mM | 832±167 | 1502±385 | 0.1525 | 0.28±0.06 | 0.42±0.11 | 0.5598 |
| 6.7 mM | 676±330 | 1280±206 | 0.0546 | 0.23±0.11 | 0.36±0.06 | 0.2945 |
| 8.7 mM | 386±80 | 1016±62 | 0.0004 | 0.13±0.03 | 0.29±0.02 | 0.0570 |

**Table S7.** Impact of Mg on ADP release. Errors are SD (N=3).

| Free Mg concentration | WT  (s^-1^) | E525K  (s^-1^) | P value  WT vs E525K |
| --- | --- | --- | --- |
| 0.20 mM | 583±80 | 834±101 | 0.0260 |
| 1.0 mM | 583±101 | 660±22 | 0.2654 |
| 2.9 mM | 434±52 | 408±69 | 0.7766 |
| 6.7 mM | 349±35 | 327±74 | 0.6677 |
| 8.7 mM | 331±66 | 279±44 | 0.3057 |

**Table S8.** Impact of Mg on basal ATPase. Errors are SD (N=3).

| Free Mg concentration | WT  ATPase  (s^-1^) | E525K  ATPase  (s^-1^) | WT  ATPase  (Relative to 0.2 mM Mg) | E525K  ATPase  (Relative to 0.2 mM Mg) |
| --- | --- | --- | --- | --- |
| 0.20 mM | 0.033±0.002 | 0.055±0.004 | 1 | 1 |
| 1.0 mM | 0.028±0.004 | 0.049±0.006 | 0.85±0.16 | 0.90±0.04 |
| 2.9 mM | 0.025±0.001 | 0.043±0.002 | 0.76±0.08 | 0.79±0.03 |
| 3.8 mM | 0.022±0.003 | 0.040±0.006 | 0.67±0.09 | 0.73±0.11 |
| 6.7 mM | 0.023±0.001 | 0.042±0.004 | 0.70±0.05 | 0.76±0.04 |
| 8.7 mM | 0.020±0.003 | 0.039±0.003 | 0.62±0.04 | 0.72±0.05 |
| Free Mg concentration | P value  WT vs E525K | P value  WT Compared to 0.20 mM Mg | P value  E525K Compared to 0.20 mM Mg | P value  WT vs E525K  (Relative to 0.2 mM Mg) |
| 0.20 mM | 0.0017 | - | - | - |
| 1.0 mM | 0.0076 | 0.0366 | 0.4481 | 0.6660 |
| 2.9 mM | 0.0001 | 0.0083 | 0.0334 | 0.7878 |
| 3.8 mM | 0.0108 | 0.0011 | 0.0079 | 0.5126 |
| 6.7 mM | 0.0016 | 0.0015 | 0.0174 | 0.1470 |
| 8.7 mM | 0.0015 | 0.0004 | 0.0056 | 0.0374 |


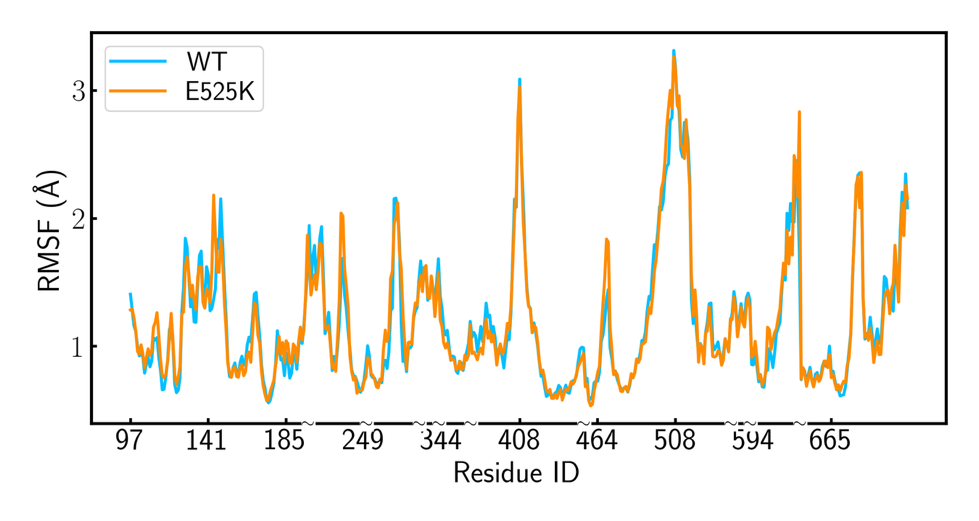


**Fig. S1.** RMSF profiles of the myosin motor domain from WT and E525K GaMD simulations. For the RMSF analysis, trajectories were aligned using motor-domain Cα atoms while excluding flexible surface loops.
